# HIV-1 reverse transcriptase subtyping revealed CRF35-AD as a current subtype in the northeast of Iran

**DOI:** 10.22099/mbrc.2025.52193.2089

**Published:** 2025

**Authors:** Zahra Mazaheri, Masoud Youssefi

**Affiliations:** 1 Department of Microbiology and Virology, School of Medicine, Mashhad University of Medical Sciences, Mashhad, Iran; 2 Antimicrobial Resistance Research Center, Mashhad University of Medical Sciences, Mashhad, Iran

**Keywords:** HIV, Phylogenetic tree, Reverse Transcriptase, Drug Resistance Mutation, Iran

## Abstract

Previously, the sequence of the HIV-1 reverse transcriptase gene was analyzed to identify mutations associated with drug resistance. We statistically analyzed the relationship between a set of additional data and increasing mutations. Existing sequences were also phylogenetically analyzed. Of all patients tested for phylogenetic tree analysis, one individual had the F subtype, two had the CRF01-AE strain, and two had the A subtype. Phylogenetic tree analysis revealed that HIV-1 CRF35-AD was the most prevalent subtype (88.6%) among the cases studied. The number of treatment discontinuations (r=0.621, df=20, *p*=0.002) and the duration of treatment (r=0.452, df=20, *p*=0.035) were significantly correlated with an increase in mutations.

## INTRODUCTION

The HIV-1 virus displays significant genetic diversity due to high error rates during transcription and the lack of proofreading by the virus's reverse transcriptase (RT). This diversity has led to the emergence of several variants. The global HIV-1 pandemic variants are categorized into subtypes A-D, F-H, and J-L. These variants can combine to create circulating recombinant forms (CRFs). As of now, 157 unique CRF types have been identified [1]. In this study, we analyzed the reverse transcription (RT) sequence to identify viral variants, obtaining CRF35-AD, CRF01-AE, and subtypes A and F. 

Most reported cases of CRF35-AD are currently found in Southeast Asia. The majority of subtype A cases are concentrated in East Africa, Central Asia, and Eastern Europe. Subtype F is predominantly observed in Latin America and Central Africa, while CRF01-AE is primarily reported in East Asia and Southeast Asia [1]. In recent years, CRF35-AD has been reported in Iran with a significantly high prevalence [2-7].

## MATERIALS AND METHODS

The procedures for sample collection, genome extraction, PCR, and sequencing have already been published. According to the previous article, RNA was extracted from the serum samples, and cDNA was synthesized. After conducting PCR, the resulting product, RT gene of HIV, was extracted from the gel and sent for sequencing. The data were analyzed using Chromas software, followed by editing with BioEdit, and subsequently examined using the HIV Drug Resistance Database [8].

In an additional investigation, the RT sequences were compared to reference genes from the NCBI Gene Bank, and the closest matches were selected. All sequences, along with the reference data, were compiled using Notepad++ and aligned using MEGA7 software. Following this, a phylogenetic analysis was conducted then a neighbour-joining tree was constructed using bootstrap replication with 1,000 iterations to generate the phylogenetic tree. The correlation between treatment duration and mutation rates, as well as the frequency of treatment discontinuation and increasing mutations, was evaluated using Pearson's correlation method.

## RESULTS AND DISCUSSION

The demographic data of the patients has been previously published. The mutation results presented in Table 1 have also been included in a prior article [8]. In this document, the same data is presented in a different format in Table 1, along with some additional information for supplementary purposes.

**Table 1:** The detected mutations against NRTI and NNRTI and the final result of each person's resistance separately for each of the drugs (The group without receiving treatment & under-treatment group).

**The group without receiving treatment**			**under-treatment group**				
**Patient number**	**NRTI Mutations**	**NNRTI Mutations**	**Patient number**	**NRTI Mutations**	**NNRTI Mutations**	**History of treatment discontinuation (times)**	**History of treatment** **(years)**
1	None	None	4	None	Y188L	0	2
2	None	Y188F	5	None	None	0	4
9	None	None	6	None	Y188H	2	8
11	None	None	7	None	Y181C	0	8
12	None	E138A	15	None	E138A	3	8
22	None	None	16	M184V, T215F	None	3	5
23	None	V179T	17	M184V	K101E, K103N, G190A, K101E+G190A	0	7
25	None	None	19	M184V	G190A	1	4
27	None	None	20	M184V, K219Q	P225H	3	9
28	None	None	21	None	E138A, G190A , P225H	0	4
32	None	None	24	None	None	0	1
33	None	None	26	None	None	3	4
36	None	None	29	None	Y188L	0	6
48	None	None	30	M184V, T215F, K219Q	None	3	8
50	None	None	31	M184V	K103E, V108I, P225H	3	3
51	None	None	35	M184V, T215F, K219Q	K101E, V106M, Y181C, G190A	3	9
53	None	Y188L	38	None	Y188L	0	3
54	None	None	40	None	None	2	1
55	None	None	42	None	None	1	1
56	None	None	43	None	None	1	2
58	None	None	57	M184V	E138A	2	7
59	None	None	60	M184V, L210W, T215Y, L210W+T215Y	None	4	8

The duration of treatment (measured in years) was significantly correlated with the increase in mutations within the treatment group (r=0.452, df=20, *p*=0.035). The number of treatment discontinuations was also significantly correlated with the number of mutations in patients (r=0.621, df=20, *p*=0.002).

It should be noted that in this study, women had a higher average CD4+ count compared to men. The average (±SD) T-CD4+ count among the women in this study was 236 (±129) cells/mm^3^, while the mean (±SD) T-CD4+ count for the men was 175 (±122) cells/mm^3^. The difference in T-CD4+ count between women and men was not statistically significant (*p*=0.161) as determined by an independent t-test. We found no significant relationship between genders and the presence or absence of mutation (*p*=0.977). Anyway, further specialized and comprehensive studies are recommended on this subject.

The data indicates that delaying the change of first-line treatment can have serious consequences, including the development of drug-resistant mutations, the risk of transmitting resistance, and ultimately, increased mortality [9]. Also, non-adherence or poor adherence to ART (Anti-Retroviral Therapy), may lead to virologic failure and the emergence of drug-resistant strains [10, 11]. Adherence even has been reported as a predictor of mortality [12]. In this study, the average length of ART treatment was 4.9 years, and the history of discontinuation was 1.4 times. Genotyping drug resistance analysis indicated several mutation in under treatment group but patients numbered 16, 20, 30, 31, 35, and 60 show a significant accumulation of mutation loads (refer to Table 1). It was revealed through their medical history that they were on first-line therapy for 6.5 years and had discontinued ART three times during treatment on average. Patients No. 16 and 31 died finally with a history of 4 years of first-line therapy and 3 times of non-adherence to ART. Furthermore, this study suggests that promoting adherence among individuals receiving ART can help optimize first-line therapy and prevent adverse outcomes caused by resistance. It's necessary to conduct genotypic drug-resistance tests every year to keep track of the patient's condition and ensure effective treatment.

**Figure 1:**
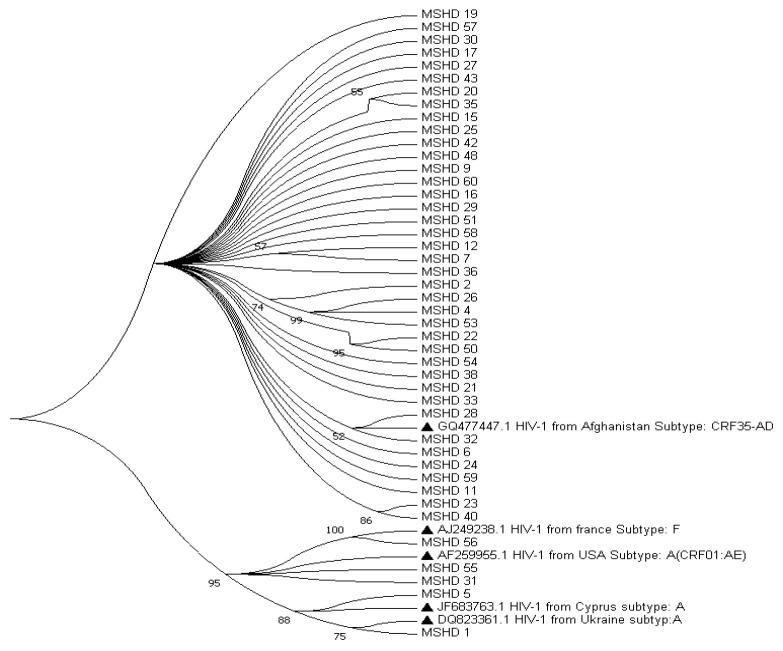
Phylogenic analysis of 44 HIV-1 Mashhad nucleotide sequences based on reverse transcriptase genes. The evolutionary analysis was inferred using the Neighbor-Joining method and a bootstrap test with 1000 replicates in MEGA7.

The results of the phylogenetic tree analysis indicated that HIV-1 CRF35-AD was the most common subtype in our cases, according to 88.6% of the cases (Fig. 1). Several studies conducted in Iran have found this to be the most prevalent subtype in the country [2-7]. 

At present, the connection between RAMs (Resistance Associated Mutations) and CRF35-AD is still unknown. Several studies suggest that mutations are similar across different subtypes [2], but some have found more mutations in CRF35-AD [4]. A few patients in this study had subtype A and CRF01-AE, and one subtype F was found among the patients. In one regional study, subtype A was the most common, while CRF01-AE [3, 4] and subtype A(2). were equally prevalent in other regions. A regional study reported the presence of HIV-1 subtype A in 5.1% of the total cases analyzed [2]. Additionally, two other studies reported CRF01-AE at rates of 3.3% and 16.7%, respectively [3, 4]. No instances of subtype F were found in any of the regional studies. During this study, two male participants died (patient No. 16 and 31). Some studies have shown that males may have a higher HIV/AIDS mortality rate than females. The reason can be considered the higher sex ratio at birth in males (resulting in a higher mortality rate), increased risky homosexual behavior in males, the better outcomes of HIV/AIDS therapy in females, the higher CD4+ levels in females during the treatment process and a better biologic response against drugs in females [13].
